# Pressure and temperature-dependent optical properties of TiTa_2_O_7_

**DOI:** 10.1039/d0ra02445g

**Published:** 2020-07-03

**Authors:** Yuxin Hu, Qiang Zhou, Liang Li, Shufan Jia, Shunli Ouyang, Tian Cui, Pinwen Zhu, Fangfei Li

**Affiliations:** State Key Laboratory of Superhard Materials, College of Physics, Jilin University Changchun 130000 P. R. China; Key Laboratory of Integrated Exploitation of Bayan Obo Multi-Metal Resources, Inner Mongolia University of Science and Technology Baotou 014010 China

## Abstract

Polycrystalline TiTa_2_O_7_ was synthesized directly by solid-state reaction methods. The structures were determined by using X-ray diffraction (XRD). The high-pressure Raman and UV-vis absorption spectra of TiTa_2_O_7_ were obtained up to 25 GPa in a diamond anvil cell (DAC) at room temperature. The Raman scattering results reveal that a pressure-induced amorphization occurs above 10.5 GPa. An inflection point was also observed at 11 GPa in the pressure-dependent bandgap energy spectra, which agrees well with the amorphization point found in Raman spectra. The temperature-dependent Raman and photoluminescence (PL) spectra of TiTa_2_O_7_ were also measured. The PL mechanism for TiTa_2_O_7_ was studied. It is worth noting that the Raman vibrations attributing to the bending vibration of the Ta (Ti)–O octahedron exhibit anomalous frequency shifts in both the high-pressure and temperature-dependent Raman spectra.

## Abstract 2

Polycrystalline TiTa_2_O_7_ entered amorphous phase at 10.5 GPa and the SEM images indicate that the porosity of the sample were decreased.

## Graphical abstract

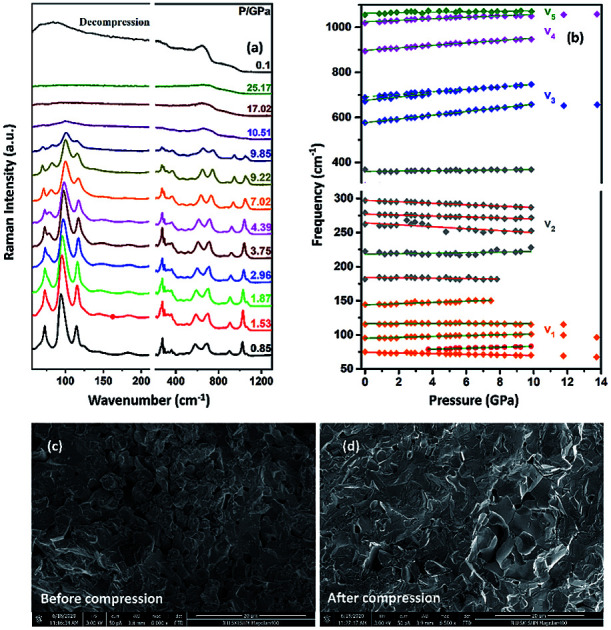

## 1. Introduction

Tantalum oxides have short metal–oxygen bond distances, high-temperature resistance and good chemical resistance, as well as excellent optical properties and expected high hardness,^1^ which have obtained worldwide attention. As a typical tantalum oxide, titanium tantalate (TiTa_2_O_7_) has a high average refractive index, relative high hardness, and high temperature resistance, these materials could be of interest for further potential applications, including synthetic gemstones, optical coatings, or low-thermal-expansion materials.^1^ In recent years, the tantalates and niobates with titanium were found to have excellent photocatalysis, for example: TiNb_2_O_7_ (ref. 2) and NaTaO_3_.^3^ Tantalate photocatalysts have unique electronic and band structure and their catalytic performance is better than other types of compounds in the decomposition of water to hydrogen, good examples are Ti–Ta alloy-based nanotubular oxide^4^ and mesoporous Ta_2_O_5_–TiO_2._ (ref. 5) TiTa_2_O_7_, the focus of our research is also considered as a promising photocatalyst.^4^ Its defects with a slightly wide band-gap under ambient conditions are expected to be modulated at high pressure.

**Fig. 1:**
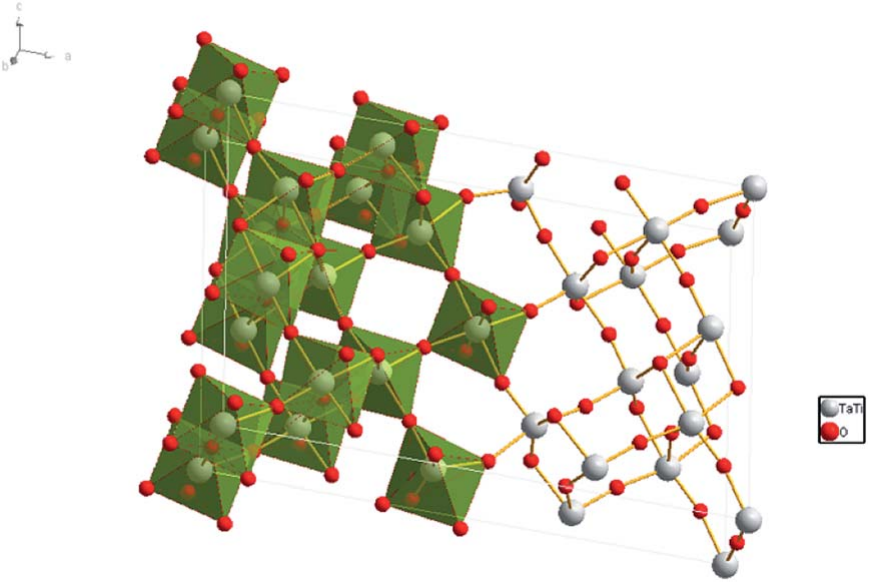
Crystal structure of TiTa_2_O_7_, the grey spheres represent the Ta and Ti atoms, red spheres represent the O atoms.

TiTa_2_O_7_ crystallizes is a monoclinic structure (space group *C*2/*m*), its lattice parameters are *a* = 20.351(3) Å, *b* = 3.801(2) Å, *c* = 11.882(2) Å, and *β* = 120.19(1)°. The structure of TiTa_2_O_7_ can be described as ‘3 × 3 shear ReO_3_’ structure, consisting of TiO_6_ octahedra sharing corners and edges (Fig. 1). This structure contains fragments of the ReO_3_ structure in the form of blocks of corner-sharing MO_6_ octahedra (M = Ti, Ta). Each of these blocks contains nine MO_6_ (3 × 3) octahedra forming linear columns along the *b*-axis. Perpendicular to the *b*-axis, the columns are connected by crystallographic shear planes. MO_6_ octahedra share edges across these shear planes.^6^ There is no ordering of the Ti^4+^ and Ta^5+^ cations among different crystallographic sites in the structure of TiTa_2_O_7_.^7^ Niobates and tantalates are isostructural with the same oxygen occupancy. Only a few studies were carried out caring about ANb_2_O_6_ (A = Fe, Mn, Mg) with columbite structure,^8–10^ and have indicated a pressure-induced volume decrease and distortion of the AO_6_ and NbO_6_ octahedra. However, the influence of the octahedral shape on properties and the monoclinic structure (TiTa_2_O_7_) under high-pressure is still unknown.

Herein, we have synthesized polycrystalline TiTa_2_O_7_, and its property is investigated using high pressure Raman spectroscopy and UV-vis absorption in DAC, as well as the temperature-dependent Raman and photoluminescence (PL). Above 10.5 GPa, monoclinic titanium tantalate started amorphization and completed above 17 GPa.

## 2. Experimental

**Fig. 2:**
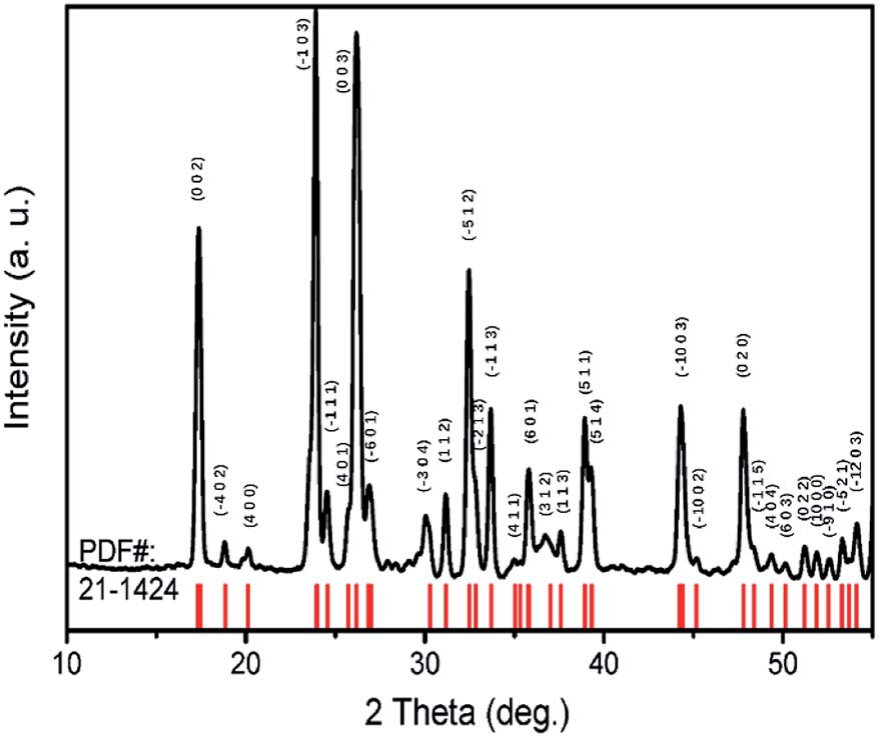
X-ray diffraction pattern of TiTa_2_O_7_ powder.

TiTa_2_O_7_ was synthesized by the conventional high-temperature solid-state reaction method. Stoichiometric amounts of TiO_2_ (Alfa Aesar, 99.995%) and Ta_2_O_5_ (Alfa Aesar, 99.99%) were mixed and ground well, the reactant mixtures were rammed and heated in alumina crucibles. The melting temperature for the TiTa_2_O_7_ is 1948 K, the pipe furnace was used to heat the mixtures gradually from 1623–1723 K with intermittent grindings. The XRD patterns of synthesized TiTa_2_O_7_ (Fig. 2) were recorded at room temperature using a Rigaku D/max-r A 12 kW X-ray diffractometer with Cu Kα radiation (*λ* = 1.5406 Å) operated at a current of 40 mA and a voltage of 40 kV. The XRD patterns fit well with the standard card (JCPDF (211424)). The result shows that the sintering product of TiTa_2_O_7_ is of high purity.

The morphology of as-prepared samples before and after compression was characterized by scanning electron microscopy (SEM, 15 kV, FEI Magellan 400).

**Fig. 3:**
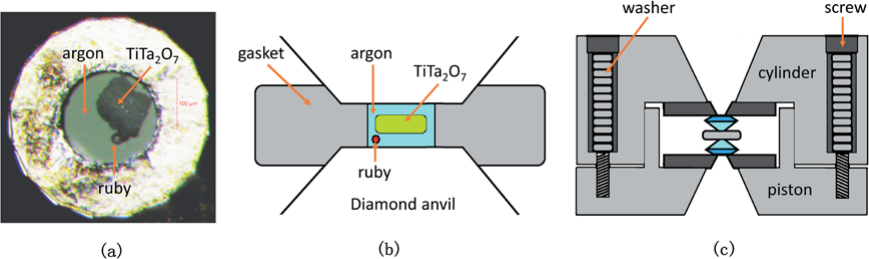
(a) The picture of sample chamber; (b) the schematic diagram of the sample chamber; (c) the schematic diagram of the DAC.

Confocal Raman spectra of TiTa_2_O_7_ were excited using the 532 nm emission line of a frequency-doubled Nd:YAG laser with a Horiba Jobin Yvon Labram-HR Evolution Raman spectrometer. The spectra were collected unpolarized under ambient conditions in a back-scattering geometry. The pressure was calibrated using the ruby fluorescence method.^11^ The T301 stainless gasket was pre-indented to a thickness of 56 μm, and a hole of 180 μm in diameter was drilled in the centre of the gasket, the TiTa_2_O_7_ powder was load in the sample chamber of DAC with the thickness of 30 μm and 70 × 150 μm in size. The schematic diagram is shown in Fig. 3. We injected argon as the pressure transmitting medium (PTM).

The temperature-dependent PL and Raman spectra were taken with a Jobin-Yvon HR800 micro-Raman spectrometer and an argon ion laser (514.5 nm) was served as the excitation source. The temperature of the sample was adjusted by the THMSE 600 Temperature Programmator (Linkam Scientific Instruments) attached to the Raman spectrometer in a range of 183.15 to 803.15 K.

The UV-vis absorption spectra were recorded using a UV-vis spectrophotometer (Perkin Elmer Lambda 850) with a synthetic IIa type diamond possessing a high transmittance in the UV region and permit photoemission studies. A deuterium–halogen (250–1000 nm) light source and an Ocean Optics QE65000 spectrometer were used as the excitation source and detector, separately.

## 3. Results and discussion

### 3.1. High-pressure Raman spectra

**Fig. 4:**
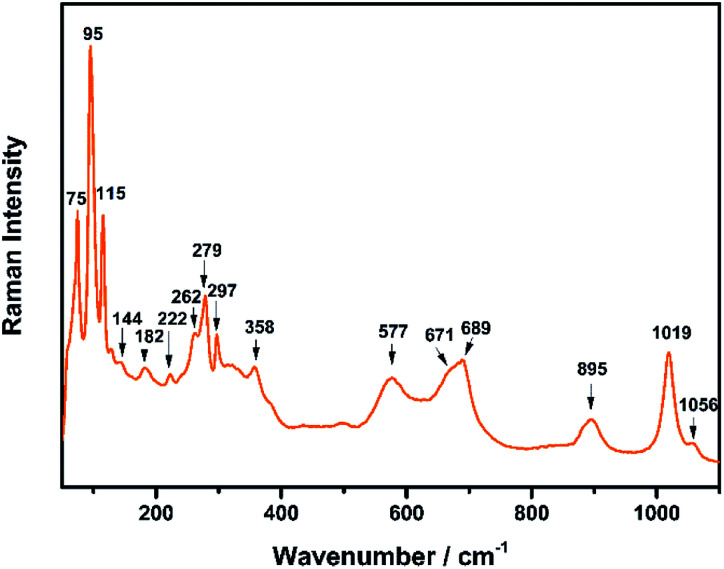
Raman spectrum of TiTa_2_O_7_ at ambient conditions.

The Raman spectrum of TiTa_2_O_7_ at ambient condition is shown in Fig. 4, and the respective Raman modes are indexed. Based on previous studies,^1,12^ we attribute the Raman peak in the low-wavenumber region (<150 cm^−1^) to external modes belonging to Ta–Ta vibrations, only metal–metal vibrations (*ν*_1_) will occur in this region. Raman modes between 150 and 400 cm^−1^ can be probably assigned to O–Ti–O or O–Ta–O symmetric and antisymmetric bending vibrations (*ν*_2_). Besides, Ti–O vibrations should be visible at lower frequencies than Ta–O vibrations, but only if the metal atoms participate in the vibration. According to Eror and Balachandran,^12^ the metal–oxygen vibrations (*ν*_3_) of the TiO_6_ octahedra occur in the wavenumber region between 550 and 700 cm^−1^, whereas two bands at 899 and 1020 cm^−1^ can be assigned to the symmetric metal–oxygen stretching vibrations (*ν*_4_) of the corner- and edge-sharing TaO_6_ octahedra, respectively.

**Fig. 5:**
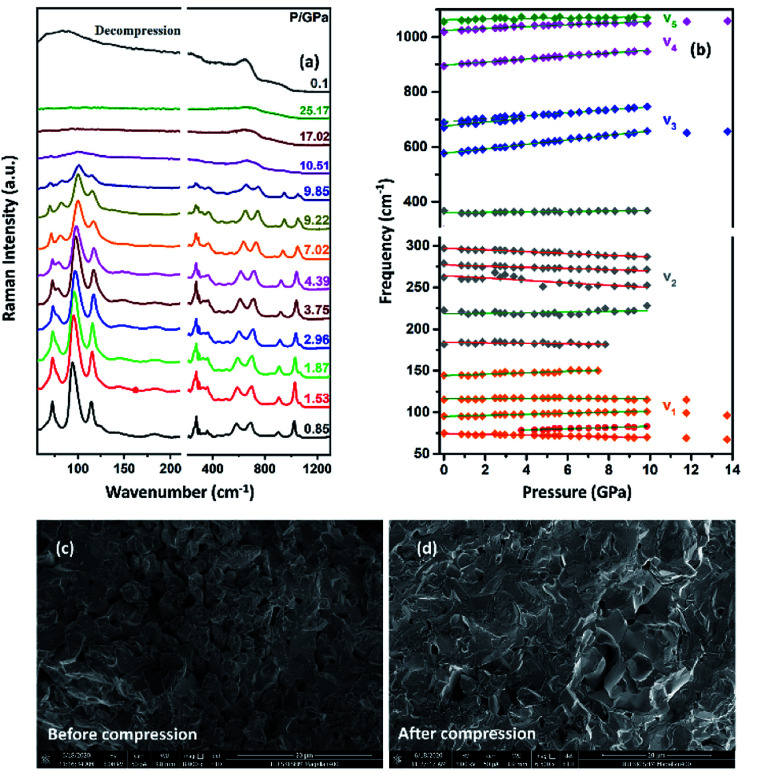
(a) High-pressure Raman spectra of TiTa_2_O_7_ and decompression Raman spectra of TiTa_2_O_7_ collected at room temperature, the gray line above is decompression spectrum to ambient condition; (b) pressure dependence of all Raman vibrations of TiTa_2_O_7_; the SEM image before (c) and after (d) compression.

To confirm the structural evolution and phase stability of TiTa_2_O_7_ under high pressure, the high-pressure Raman spectra of TiTa_2_O_7_ were performed up to 25.17 GPa. For comparison, representative Raman spectra of TiTa_2_O_7_ at various pressures upon compression and decompression are shown in Fig. 5a. In low-pressure realm, little change is observed until 3.75 GPa, where the 75 cm^−1^ mode split into two peaks. Above 10.5 GPa, the original peaks from the monoclinic TiTa_2_O_7_ collapsed into two very broad bands at about 100 and 650 cm^−1^, indicating the pressure-induced amorphization was taken place. After releasing the pressure, no Raman mode was recovered, which implies that the pressure-induced transformation was completely irreversible. These results were confirmed by multiple experiments with different compressing rates, no difference was found when we retain pressure in a relatively long period. While we change the power of the laser, the wavenumber of titanium tantalate didn't suffer a significant change, the intensity of the peak went with corresponding laser power with a certain coefficient.

To further understand the high-pressure behavior of TiTa_2_O_7_ under high pressure, Raman frequencies of TiTa_2_O_7_ as a function of pressure are plotted in Fig. 5b, and the corresponding data are shown in Table 1. It is found that most of the vibration modes shift to higher frequencies with pressures until 10.51 GPa. Usually, the vibrational mode frequencies are expected to increase as bonds are compressed. However, 182 cm^−1^, 162 cm^−1^, 279 cm^−1^, and 297 cm^−1^ modes of *ν*_2_ are found to shift towards lower wavenumbers with increasing pressure. The existence of such soft modes indicates the elongation of the O–Ta–O/O–Ti–O bond under pressure, as well as the structural instability of TiTa_2_O_7_ upon compression. This is because although the force constant of the O–Ta–O/O–Ti–O bond is smaller than that of Ta–O bond, the effect of pressure on Ta–O bond is mainly reflected in the bond length, while for the O–Ta–O/O–Ti–O bond, the pressure changes the bond angle. The four modes red-shifted and intensity increased slightly as the pressure increasing. It can be explained that the O–Ta–O and O–Ti–O bonds become longer.

**Table 1:** Frequency (*ν*) assignments and its pressure derivative (d*ν*/d*P*) and temperature derivative (d*ν*/d*T*) for the various Raman modes of TiTa_2_O_7_ (ref. 1 and 12)

Mode number	Mode assignment	*ν*/cm^−1^	d*ν*/d*P*	d*ν*/d*T*
*ν*_1_	Ta–Ta bond	75	−0.46	0.002
		95	0.63	0.003
		115	0	0.005
		144	0.87	−0.006
*ν*_2_	O–Ta–O or O–Ti–O bond	182	−0.27	0.007
		222	0.40	−0.007
		262	−1.43	−0.008
		279	−0.71	0.002
		297	−1.07	0.002
		358	0.77	0.018
*ν*_3_	Ti–O bond	577	8.20	0.014
		671	7.38	0.018
		689	5.47	0.024
*ν*_4_	Ta–O bond	895	5.52	0.011
		1019	3.27	0.020
*ν*_5_	Not known	1056	1.02	0.026

The SEM images of TiTa_2_O_7_ were taken before and after the compression. It could be clearly observed that the porosity of the sample were decreased after it was recovered to ambient conditions, due to the relative high hardness, no significant change of grain size were found.

**Fig. 6:**
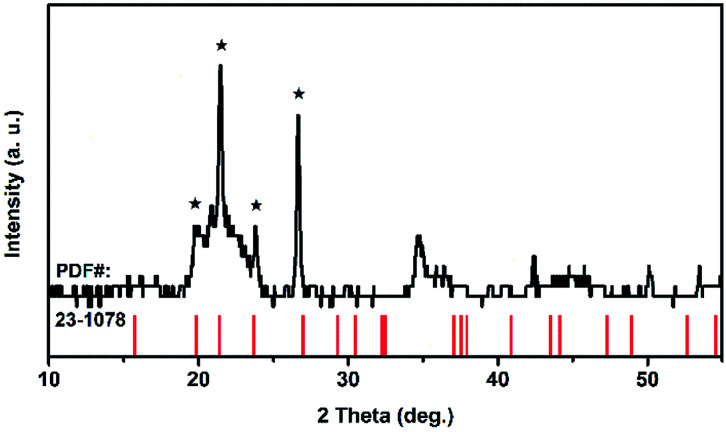
XRD pattern of the TiTa_2_O_7_ after compression.

Because of its high symmetry, TaO_6_ octahedra has only one independent bond angle, and the other bond angles change with this independent one.^13^ It can be considered that the shortening of six Ta–O bonds in different degrees is accompanied by the change of O–Ta–O/O–Ti–O bond, moreover, the bond length of Ta–Ta, corresponding to the Raman located at 75 cm^−1^, became longer as the pressure increased, in other words, the distance between the two TaO_6_ octahedra became further, while the volume of the TaO_6_ octahedra became smaller, which leads to the distortion and deformation of bonds between the TaO_6_ octahedra. Eventually, the TiTa_2_O_7_ framework ultimately collapsed into the amorphous state. The XRD pattern were measure after decompression as shown in Fig. 6, the characterize peak of TiTa_2_O_7_ were disappeared and the remaining peak fit the standard card of TiO (JCPDF (231078)) well, indicating that the sample entered the amorphous phase and the transition is irreversible.

### 3.2. UV-vis absorption spectra under high pressure

To shed light on the complex pressure-induced order–disorder processes occurring in TiTa_2_O_7_ and to understand the relationship between structure and optical properties, UV-vis absorption spectroscopy was performed and focused on the explanation of the strong nonlinear pressure dependence of the direct bandgap energy in TiTa_2_O_7_ at relatively low pressures.

**Fig. 7:**
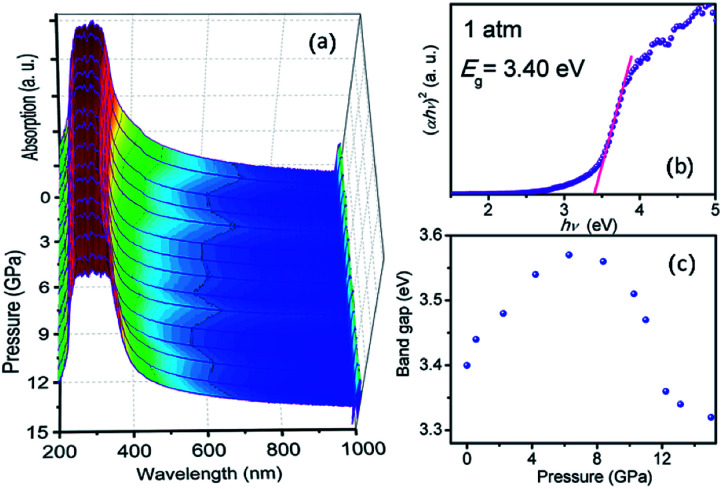
(a) Pressure dependent UV-vis absorption spectra of TiTa_2_O_7_; (b) the variations in (*αhν*)^2^*versus* photon energy (*hν*) of the TiTa_2_O_7_ at 1 atm; (c) band-gap of TiTa_2_O_7_ as a function of pressure.

Fig. 7a plots the pressure-dependent absorption spectra of TiTa_2_O_7_. The TiTa_2_O_7_ has a low absorption coefficient (*α*) (about 0.25 cm^−1^) in the wavelength range from 1000 to 425 nm, representing the transparent region. As for the ultraviolet region (<425 nm), the crystal has heavy absorption.

Photon energy *hν* can depend on the absorption coefficient by the following eqn (1):^14,15^

1 *αhν* = (*hν* − *E*_g_)^*k*^

where *k* can be 1/2, 2, 3/2, or 3 to allow direct, indirect, forbidden direct, and indirect transitions, respectively. For the TiTa_2_O_7_, the best *k* fitted to be 1/2, which demonstrates that TiTa_2_O_7_ is a direct transition material, and it also agrees well with previous theoretical results.^1^ The band-gap energy was achieved to be 3.40 eV by extrapolating the linear region of (*αhν*)^2^*vs. hν*, as shown in Fig. 7b, which fits well with previous study.^2,4^

The sample maintained a relatively clear absorption edge within the pressure range of 0–12 GPa. It can be observed that the direct bandgap energy of TiTa_2_O_7_ exhibits a strong non-linear pressure dependence up to 15 GPa. Therefore, we infer that pressure leads to the valence band maximum (VBM) and the conduction band minimum (CBM) change of the band structure. Generally, upon compression, the lattice constant and interatomic distances become smaller, the wave functions become more overlapping, this led to the broadening of the bandwidth, as a result, the band-gap became narrower.^16^ From the high-pressure Raman spectrum of TiTa_2_O_7_, the elongation of the O–Ta–O/O–Ti–O bond with increasing pressure is a good fit for the fact that in Fig. 7c, the direct bandgap energy in TiTa_2_O_7_ increases within the range 0–7.3 GPa, and decrease after 7.3 GPa. It can be explained that under 10.5 GPa, the volume of the TaO_6_ octahedra decreases gradually, and the interatomic spacing corresponding to the vibration mode in the high wavenumber region became smaller with the increase of pressure. And there is an inflection point in the decreasing trend at 11 GPa, similarly, the previous Raman spectroscopy shows that above 10.51 GPa, the amorphous phase transition of TiTa_2_O_7_ began to take place.

Our results indicate that the nonlinear pressure dependence of the direct bandgap energy is related to the bending vibration of *ν*_2_ mode at high pressure, the inflection point at 11 GPa could be mainly ascribed to the effect of TiTa_2_O_7_ amorphization.

### 3.3. Temperature-dependent Raman spectra

**Fig. 8:**
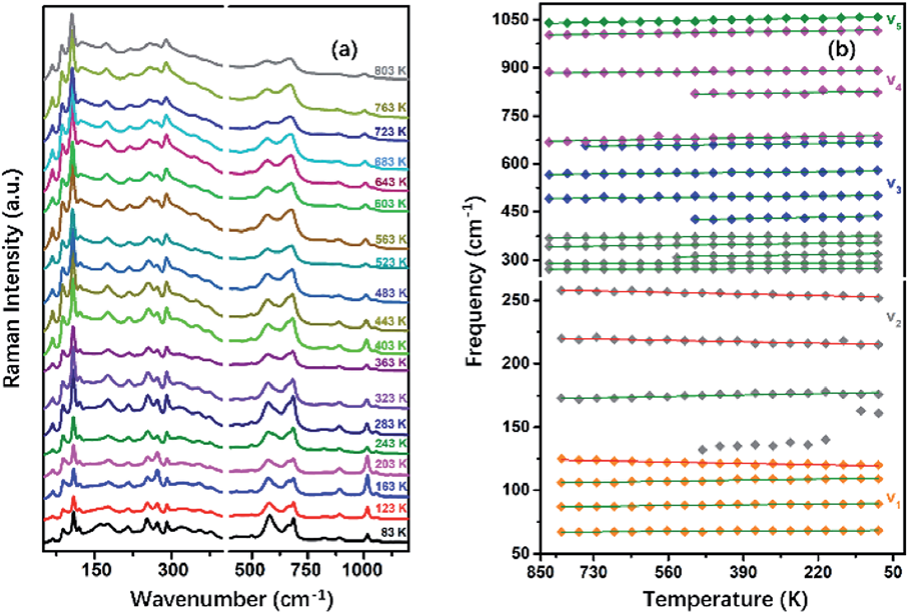
(a) Raman spectra TiTa_2_O_7_ at different temperatures; (b) peak shift diagram and three abnormal frequency shifts of Raman frequency at the variable temperature of TiTa_2_O_7_.

To understand the variable-temperature behavior in TiTa_2_O_7_ at ambient pressure, *in situ* temperature-dependent Raman measurement of TiTa_2_O_7_ crystal was carried out in temperature range from 83.15 to 803.15 K. The spectra of TiTa_2_O_7_ was collected every 42 K. The Raman spectra of all temperature points are shown in Fig. 8a. It can be seen from the spectra that with the increase of temperature, the Raman peak shifts to lower wavenumber, and the peak width widens continuously. No sudden change of peak position or new Raman peak can be observed, and there is no phase transition occurs in the whole temperature range. With the increasing temperature, the thermal motion of atoms in the polymer increases gradually, while the force between atoms decreases, which leads to the Raman peaks shift towards lower wavenumber. The irregular thermal motion of atoms is the reason for abnormal vibration of each bond together with the decrease of molecular structure order, which finally contributes to the gradual broadening of the superposition of similar Raman vibration frequencies.

Former research indicates that, at higher temperatures, the maximum thermal expansion occurs perpendicular to the endless linear columns and zigzag chains of the corner- and edge-sharing octahedra, respectively. Along the crystallographic *b*-axis, negative thermal expansion values can be partly observed in the previous study.^1^ Raman mode frequencies of TiTa_2_O_7_ as a function of temperature are plotted in Fig. 8b, and the corresponding data are shown in Table 1. In Fig. 8b, the Raman peaks 222 cm^−1^ and 262 cm^−1^ modes of *ν*_2_ are found to shift towards lower wavenumbers with the decrease of temperature, this may be related to the bending vibration of *ν*_2_ mode at high pressure. We have reason to believe that the negative thermal expansion of TiTa_2_O_7_ along the *b*-axis at a high temperature can be explained by the change of the bond angle of the O–Ta–O/O–Ti–O bonds by the external force under the condition of increasing pressure or decreasing temperature.

### 3.4. Temperature-dependent PL spectra

**Fig. 9:**
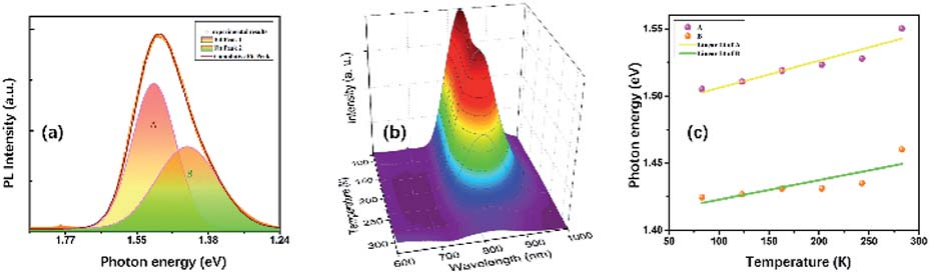
(a) Photoluminescence spectra of TiTa_2_O_7_ and its calculation fitting at 83.15 K; (b) temperature-dependent PL spectra of TiTa_2_O_7_; (c) the temperature-dependent PL peak positions.

At ambient conditions, the PL of TiTa_2_O_7_ is relatively weak compared with other materials, so no PL could be seen when it was placed in a DAC. Generally speaking, decreasing temperature and increasing pressure may induce a similar effect on studied samples, thus the temperature depend PL was collected here. Fig. 9a shows the PL spectrum of TiTa_2_O_7_ at 83.15 K, the PL peak can be well fitted (Gaussian profile) to two emission peaks which centred at 1.42 eV and 1.51 eV. Notably, the PL emission centres at 1.42–1.51 eV and instead of the obtained bandgap (∼3.4 eV) from TiTa_2_O_7_ excited by using a 514 nm laser line at 83.15 K, this phenomenon cannot be attributed to the normal band-to-band transition. Since the electronic configuration of TiTa_2_O_7_ is the same as that of niobate–oxygen octahedral, its luminescence mechanism is consistent with that of TiTa_2_O_7_. In the case of PbMg_1/3_Nb_2/3_O_3_–PbIn_1/2_Nb_1/2_O_3_ systems (PMN–PIN), the special luminescence tend to associate the origin of PL bands with the Nb–O systems without the influence of Mg and Pb.^17^

The two fitting PL peaks at 1.42 and 1.51 eV of TiTa_2_O_7_, consistent with the A peak (with a stronger intensity and higher energy) and B peak (with a weaker intensity and lower energy), which are mainly determined by the defect and regular Ta–O respectively. To understand the observed phenomenon, we have to consider the non-radiative transition of the exciton. The photo-excitation allows electrons (e^−^) in the oxygen states above the valence band (V_o_) to the conduction band (CB) and leaving holes in the V_o_, and then the excited (e^−^) in the CB relaxes to intrinsic Ta_i_ after a non-radiative transition process. Eventually, the relaxed (e^−^) recombines with holes cause the PL emission. There would be two types possible in this case, one is the B peak, another is the A peak which is led by defect states. Therefore, the defect emission A is from the e^−^ recombines with holes directly. While the B procedures an emission and vibration, relax photon and phonon.

The temperature dependence of TiTa_2_O_7_ PL is shown in Fig. 9b, the intensity decreased when the *in situ* temperature rose. The PL intensity is sensitive to temperature and can be easily affected. The rise of temperature often leads to a decrease in PL intensity, the main reason is the internal energy conversion of the molecule. As the temperature increased, the molecular thermal motion became more intense, resulting in weaker interatomic bonding and electronic structure became distorted, all of these consequences led to fluorescence quenching. And the temperature-dependent PL emission can also be fitted by Gaussian to two peaks. The temperature-dependent value of the two peak centres from 83 to 283 K is plotted in Fig. 9c. As the temperature increased, both of the two centres blue-shifted and we fitted the two data linearly. The shift speed of defect A bonds (1.99 × 10^−4^ eV K^−1^) is slightly faster than the B bonds (1.45 × 10^−4^ eV K^−1^). The blueshifts can be attributed to the shrinkage of Ta_i_–CB and V_o_–VB, and the result of the broaden of recombining energy between Ta_i_ and V_o_.

## 4. Conclusions

The optical properties of TiTa_2_O_7_ under high pressure and different temperature are reported in this article respectively. In the high-pressure Raman spectra, the TiTa_2_O_7_ was amorphized at 11 GPa. The anomalous frequency shift in the high-pressure and temperature-dependent Raman spectra, as well as the nonlinear pressure dependence of the direct bandgap energy can be attributed to the bending vibration of the O–Ta–O and O–Ti–O bonds in the Ta (Ti)–O octahedra and negative thermal expansion coefficient along the *b*-axis of the crystal structure. The PL spectra of TiTa_2_O_7_, attributed to the TaO_6_ octahedron, are also presented. These results are very important for understanding the high-pressure and low-temperature behaviors in tantalates for their applications in extreme environments.

## Conflicts of interest

There are no conflicts to declare.

## Supplementary Material
